# Post-translational regulation of endothelial nitric oxide synthase in vascular endothelium

**DOI:** 10.3389/fphys.2013.00347

**Published:** 2013-12-13

**Authors:** Jin Qian, David Fulton

**Affiliations:** ^1^Pulmonary and Critical Care, School of Medicine, Stanford University/VA Palo Alto Health Care SystemPalo Alto, CA, USA; ^2^Vascular Biology Center, Georgia Regents UniversityAugusta, GA, USA

**Keywords:** eNOS, nitric oxide, post-translational, vessel, vascular diseases

## Abstract

Nitric oxide (NO) is a short-lived gaseous signaling molecule. In blood vessels, it is synthesized in a dynamic fashion by endothelial nitric oxide synthase (eNOS) and influences vascular function via two distinct mechanisms, the activation of soluble guanylyl cyclase (sGC)/cyclic guanosine monophosphate (cGMP)-dependent signaling and the S-nitrosylation of proteins with reactive thiols (S-nitrosylation). The regulation of eNOS activity and NO bioavailability is critical to maintain blood vessel function. The activity of eNOS and ability to generate NO is regulated at the transcriptional, posttranscriptional, and posttranslational levels. Post-translational modifications acutely impact eNOS activity and dysregulation of these mechanisms compromise eNOS activity and foster the development of cardiovascular diseases (CVDs). This review will intergrate past and current literature on the post-translational modifications of eNOS in both health and disease.

Cardiovascular disease (CVD) remains the primary cause of death in developed and developing countries, and almost 800,000 people die annually in the US from CVDs that include atherosclerosis, hypertension, congestive heart failure, and stroke (Heron et al., 2009). The endothelium is a single layer of cells lining the lumen of all blood vessels. Endothelial cells provide a barrier to thrombosis, regulate both acute, and chronic blood flow, local inflammation, and vascular cell proliferation (Cines et al., 1998). Loss of endothelial function precedes vascular disease and is thought to be an initiating event (Jensovsky, 1994). Nitric oxide (NO) is a major mediator of endothelial function and is synthesized in endothelial cells by endothelial nitric oxide synthase (eNOS). eNOS-derived NO plays a vital role in maintaining cardiovascular homeostasis by influencing vascular tone, smooth muscle cell proliferation, and migration, leukocyte adhesion, and platelet aggregation (Forstermann and Munzel, 2006). Numerous studies have shown that eNOS is protective against pathologic vascular remodeling, hypertension and atherosclerosis (Shesely et al., 1996; Rudic et al., 1998; Kuhlencordt et al., 2001). Moreover, reduced expression and dysregulation of eNOS which result in the decreased bioaviability of NO and the increased production of superoxide instead of NO, increases the severity of CVD (Oemar et al., 1998; Ozaki et al., 2002). Therefore, corruption of eNOS/NO signaling is considered an early and common mechanism underling numerous vascular pathologies. A greater understanding of eNOS regulation and new approaches to improving eNOS function is a vital goal in the improved treatment of CVDs.

## NO

In 1980, an endothelium-derived relaxing factor (EDRF) was discovered in rabbit aortae by Furchgott and Zawadzki (1980). Breakthrough studies by several groups later identified EDRF as NO (Katsuki and Murad, 1977; Ignarro et al., 1987; Palmer et al., 1987). NO is a highly lipophilic, hyper reactive, diffusible free radical gas (Dudzinski et al., 2006) with a short half-life in biological fluids (Thomas et al., 2001). NO is produced in vary degrees in the cardiovascular, nervous, digestive and immunological systems where it exerts a variety of biological actions under both physiological and pathological conditions (Bian et al., 2008). The paired oxygen and nitrogen atoms of NO exhibit characteristics of both a partial double bond and partial triple bond as a result of an unpaired electron (Dudzinski et al., 2006). The free radical character of NO confers unique reactivities and is responsible for the interaction of NO with numerous cellular and extracellular targets. As a lipophilic gas, NO readily diffuses away from the site of synthesis, across multiple cellular membranes to alter signaling in distal cells (Dudzinski et al., 2006). Well-characterized actions of NO include the stimulation of vasodilation, inhibition of smooth muscle cell proliferation, leukocyte adhesion, and platelet aggregation (Forstermann and Munzel, 2006). Impaired NO activity is commonly observed as a critical event in the pathogenesis of CVD (Verhaar et al., 2004; Dudzinski et al., 2006; Versari et al., 2009). As a consequence, a major therapeutic goal in improving endothelial function in CVD is centered around enhancing deficient NO-signaling (Verhaar et al., 2004).

## NOS

The NOS family of enzymes consists of three distinct isoforms: neuronal (nNOS, alternatively designated NOSI as it was the first NOS isoform to be discovered), inducible (iNOS or NOSII), and endothelial (eNOS or NOSIII) NOS. All three isoforms are expressed in the human cardiovascular system (Balligand et al., 1994; Shen et al., 1999; Sears et al., 2003). The neuronal isoform, nNOS has been shown to be expressed in vascular smooth muscle of certain types of blood vessels (Forstermann and Sessa, 2012). The inducible, iNOS is not normally present in blood vessels but can be induced following infection or cytokine stimulation and is prominently found in vascular smooth muscle and immune cells (Kroncke et al., 1998; Kibbe et al., 1999). In contrast, eNOS is prominently expressed in all endothelial cells (Toda, 2012). Expression of eNOS was originally thought to be constitutive, but recent studies have shown that its expression levels fluctuate in response to mechanical stimulation (Ziegler et al., 1998), growth factor (Ziegler et al., 1998; Bouloumie et al., 1999), and cytokines (Gomez-Fernandez et al., 2005).

eNOS is a bi-domain enzyme comprising a C-terminal reductase domain which binds nicotinamide adenine dinucleotide phosphate (NADPH), the flavins mononucleotide (FMN), and flavin adenine dinucleotide (FAD); an N-terminal oxidase domain which binds heme, zinc, tetrahydrobiopterin (BH4), and calmodulin. In addition to the bi-domain catalytic structure, eNOS forms homodimers and dimerization is essential for enzymatic activity (Panda et al., 2002). Electrons flow from the C-terminal reductase domain of one NOS monomer to the N-terminal oxygenase domain of the other NOS monomer (Siddhanta et al., 1998). The primary mode of enzyme activation is the binding of calcium-bound calmodulin to the N-terminal CaM-binding domain. This facilitates a structure change and the flow of electrons from NADPH through the flavins to the oxygenase domain of the other eNOS monomer (Abu-Soud et al., 1994). Within the oxygenase domain, molecular oxygen is bound to heme and reduced and then incorporated into L-arginine to form NO and L-citrulline (Fleming and Busse, 1999; Verhaar et al., 2004). To generate NO, 1 mole of l-arginine, 1.5 moles of NADPH, and 2 moles of molecular oxygen are required (Dudzinski et al., 2006). To efficiently produce NO, eNOS must effectively coordinate the binding of multiple substrates and cofactors. Dirsuption of this highly coordinated catalysis, such as which occurs in the absence of adequate substrate concentrations or other modifications can result in the production of superoxide and peroxynitrite (Dudzinski et al., 2006).

## Functional mechamisms of NO

In blood vessels, NO signaling is orchestrated via at least two distinct mechanisms. The first is the well characterized activation of the high affinity soluble guanylyl cyclase (sGC)-cyclic guanosine monophosphate (cGMP) signaling pathway (Ziche et al., 1993; Dimmeler et al., 1997; Ziche and Morbidelli, 2000; Friebe and Koesling, 2003) This pathway has been established to mediate the NO-dependent relaxation of vascular smooth muscle and the ability of NO to suppress platelet aggregation (Friebe et al., 2007). NO-sensitive soluble guanylyl cyclase (sGC) is the cognate receptor for NO and once activated, sGC catalyzes the formation of the intracellular messenger cGMP. The affinity of sGC for NO is very high and thus low amounts of NO (nM) activate this pathway (Beckman and Koppenol, 1996). Binding of NO, to the reduced heme moiety of sGC increases the conversion of guanosine triphosphate (GTP) to cGMP, which in turn activates downstream effector systems such as protein kinases, phosphodiesterases, and ion channels (Murad, 1986). Dysfunction of this pathway has been reported to contribute to the pathogenesis of many disorders, including hypertension and atherosclerosis (Ruetten et al., 1999; Mizuno et al., 2010). Genetic deletion of sGC results in reduced endothelial-dependent relaxation, reduced ability of NO to relax smooth muscle and prevent platelet activation and hypertension (Buys et al., 2008; Dangel et al., 2010; Groneberg et al., 2010).

However, not all actions of NO are dependent on activation of sGC and cGMP/PKG signaling. A second mechanism is called S-nitrosylation and involves the ability of NO or its metabolites to react with cysteine residues of target proteins (Stamler et al., 2001; Dudzinski et al., 2006). In the past decade, reversible redox modifications of cysteine residues have garnered considerable attention as a mechanism of intracellular signaling. S-nitrosylation is increasingly recognized for its ability to influence protein function in a reversible manner analogous to phosphorylation (Stamler et al., 2001). Indeed, like phosphorylation, a motif for S-nitrosylation has been postulated that consists of a cysteine residue located between an acidic and a basic amino acid that together lie within a hydrophobic environment (Yeh et al., 1999; Hess et al., 2001, 2005; Zimmet and Hare, 2006; Foster et al., 2009a; Xue et al., 2010). The reversal of S-nitrosylation has been shown to be mediated by two major enzymes, the S-nitrosoglutathione reductase (GSNOR) (Liu et al., 2001, 2004) and thioredoxin 1 (Trx1) (Mitchell and Marletta, 2005). S-nitrosylation has been shown to impact a wide range of biological processes including apoptosis (Dimmeler et al., 1997; Kang-Decker et al., 2007; Benhar et al., 2008; Cho et al., 2009), cellular trafficking (Ozawa et al., 2008), proliferation (Ignarro et al., 2001), NO synthase activity (Erwin et al., 2005), ion channel activity and muscle contractility (Xu et al., 1998), transcription factor activity (Palmer et al., 2000), protein secretion (Matsushita et al., 2003), blood flow (Singel and Stamler, 2005), as well as a wide range of pathophysiological conditions (Foster et al., 2009b). The dysregulation of protein S-nitrosylation has been observed in a wide spectrum of human diseases, and is increasingly recognized as source of aberrant cellular function (Lim et al., 2008; Cho et al., 2009; Lima et al., 2010; Wei et al., 2010; Seth and Stamler, 2011). In contrast to sGC signaling, higher amounts of NO (μM) are required for nitrosylation and because of the high diffusion co-efficient of NO (Martinez-Ruiz and Lamas, 2005), this may allow for the selective nitrosylation of proteins within close proximity to the source of NO. To date, a large number of SNO-proteins have been identified, but the observed specificity of S-nitrosylation in terms of target proteins and specific cysteines within modified proteins is not yet well understood (Seth and Stamler, 2011).

## Post-translational regulation of eNOS

eNOS activity and ultimately the amount of NO synthesized is controlled by a complex integration of transcriptional, post-transcriptional and post-translational mechanisms (Dudzinski et al., 2006). Acutely, eNOS activity can be robustly regulated by a number of post-translational modifications, including fatty acid acylation, substrate, and co-factor availability, degree of phosphorylation, S-nitrosylation, acetylation, and protein-protein interactions.

### Intracellular localization

Within endothelial cells, eNOS has been shown to be concentrated within plasma membrane (PM) caveolae, a pocket-like invagination on the membrane, which is enriched in cholesterol and sphingolipids and is important for signal transduction (Lisanti et al., 1994). Not surprisingly, it has been shown that caveolae are important for eNOS function (Shaul et al., 1996; Sowa et al., 2001). The extraction of membrane cholesterol and exposure of endothelial cells to oxidized low density lipoprotein (LDL) all have been shown to reduce eNOS activity by displacing eNOS from the PM to intracellular sites (Blair et al., 1999; Nuszkowski et al., 2001) suggesting that eNOS targeting to cholesterol enriched domains is important for NO synthesis. The subcellular location of eNOS is mediated by protein fatty acid acylation. There are two major lipid modifications: the co-translational N-myristoylation on glycine-2 and post-translational palmitoylation on cysteines-15 and 26. Myristoylation is the first and necessary step for subsequent palmitoylation (Liu and Sessa, 1994; Robinson and Michel, 1995). Once eNOS is myristoylated and palmitoylated, it is subsequently targeted to the Golgi complex and plasmalemmal caveolae (Liu and Sessa, 1994; Liu et al., 1995, 1997; Garcia-Cardena et al., 1996b). Previous studies have shown that a glycine-2 to alanine (G2A) mutant of eNOS is neither myristoylated nor palmitoylated and can be found in the cytosol instead of bound to peripheral membranes. The G2A eNOS retains equivalent catalytic activity in assays replete with cofactors, but within cells, it produces less NO than the wild type eNOS. These studies revealed an important role of intracellular location in the catalytic regulation of eNOS (Church and Fulton, 2006). The importance of palmitoylation for optimal eNOS function was revealed by mutation of Cys-15 and Cys-26 to serines, which prevents eNOS palmitoylation. Loss of palmitoylation leads to the intracellular redistribution of eNOS and diminishes NO synthesis in cells (Liu et al., 1995; Robinson and Michel, 1995). Further analysis of eNOS targeting motifs revealed that the first 35 amino acids including the N-myristoylation and palmitoylation sites are sufficient to provide intracellular targeting of eNOS to regions of the Golgi and PM (Liu et al., 1997). In addition to the PM, eNOS can be found attached to various intracellular membranes, such as Golgi, which produce considerably less NO than PM eNOS. Functional relevance of eNOS on the Golgi remains to be further established (Liu et al., 1997). The appropriate intracellular localization and distribution of eNOS in PM and Golgi apparatus is necessary for Akt (and agonist)-dependent eNOS phosphorylation on Ser-1179 and is impaired in the cytosolic G2A eNOS (Fulton et al., 2002). In COS-7 cells, when reconstituted with a PM localized eNOS, eNOS is highly phosphorylated and highly active in response to the elevation of intracellular calcium. In contrast, Golgi eNOS is less phosphorylated under basal conditions, but preferentially activated via mechanisms involving Akt-dependent phosphorylation (Fulton et al., 2004). It has also been demonstrated that eNOS and caveolin-1 can translocate into the nucleus following vascular endothelial growth factor (VEGF) stimulation (Feng et al., 1999). The presence of eNOS in mitochondria has also been shown and termed as “mitochondria NOS” (mtNOS), which is thought to contribute to superoxide production in endothelial cells (Brodsky et al., 2002). C-terminal polybasic domains with an autoinhibitiory domain of eNOS have also been shown to influence membrane binding and mitochondrial localization (Gao et al., 2004). However, the targeting of eNOS to the nucleus or mitochondria results in an enzyme that produces very little NO (Jagnandan et al., 2005). Targeting calcium-independent forms of NOS (iNOS) or a novel calcium-insensitive eNOS to the cytosol or to the nucleus and mitochondria resulted in activity equal to that targeted to the membranes of the Golgi and PM suggesting that calcium or mechanisms regulating calcium-sensitivity are central to location-dependent changes in eNOS activity (Jagnandan et al., 2005; Church and Fulton, 2006).

Although location is clearly an important factor for regulating eNOS activity, much less is known about its contribution to downstream NO-dependent signal transduction. Recent studies have found that eNOS in the Golgi can influence the S-nitrosylation of local proteins (Iwakiri et al., 2006; Sangwung et al., 2012). When expressed in the endothelium of intact blood vessels, the PM location of eNOS results in a greater ability to elicit cGMP-dependent signaling and endothelium-dependent relaxation vs. a Golgi-targed enzyme (Qian et al., 2010). The ability of PM eNOS to elicit more pronounced endothelium-dependent relaxations and greater increases in cGMP accumulation is not surprising given the extraordinary sensitivity of sGC for NO (Russwurm et al., 1998) and most likely reflects the increased NO production from this location (Fulton et al., 2004). Consistent with these studies, the S-nitrosylation-dependent inhibition of Von Willebrand factor (vWF) release from endothelial cell is greater in endothelial cells expressing eNOS at the PM compared to the Golgi. Mechanistically, it was shown that the amount of NO, and not the location of synthesis, is the most important variable influencing protein S-nitrosylation and vWF suppression (Qian et al., 2010). The importance of higher concentrations of NO are also observed in the ability of either PM or Golgi-restructed eNOS to influence inflammatory NF-κ B signaling (Qian and Fulton, 2012). While a PM location favors the highest output of NO from eNOS, it is also the most susceptible to extracellular influences such as oxidized LDL which selectively reduces NO release from PM-targeted eNOS (Shaul, 2002; Zhang et al., 2006). eNOS restricted to the Golgi is resistant to the actions of oxidized LDL and Golgi restricted eNOS is also capable of supplying biologically active NO to adjacent smooth muscle cells and mediating endothelium-dependent relaxation. While eNOS is generally regarded as being protective in murine models of atherosclerosis (Kuhlencordt et al., 2001), it is not yet known whether a Golgi location of eNOS would offer more protection against lesion formation vs. the PM.

### eNOS phosphorylation

eNOS is dynamically regulated by changes in protein phosphorylation. It is known that eNOS can be phosphorylated at multiple sites, including serine (S), threonine (T), and tyrosine (Y) residues (Michel et al., 1993; Corson et al., 1996; Garcia-Cardena et al., 1996a; Fulton et al., 2005). Seven primary sites of eNOS phosphorylation have been identified in human isoform on Y81, S114, T495, S615, S633, Y657, and S1177 (equivalent to Y83, S116, T497, S617, S635, Y659, and S1179 of bovine eNOS due to two extra amino acids in the bovine eNOS sequence) (Venema, 2002; Fulton et al., 2005; Fisslthaler et al., 2008). Folic acid incubation can modulate eNOS phosphorylation at multiple sites without change eNOS expression level (Taylor et al., 2013).

The phosphorylation of human eNOS S1179 on the C-terminal reductase domain was one of the first eNOS phosphorylation sites identified and is a positive regulator of eNOS activity (Fulton et al., 1999; Scotland et al., 2002). Phosphorylation of S1179 via Akt has been shown to be important in the activation of eNOS in endothelial cells in response to VEGF and shear stress (Dimmeler et al., 1999; Fulton et al., 1999; Gallis et al., 1999; Michell et al., 1999). Other protein kinases have also been shown to phosphorylate eNOS at S1179, including adenosine monophosphate-activated kinase (AMPK) (Chen et al., 1999), CaM protein kinase II (Fleming et al., 2001), protein kinase A (PKA) (Butt et al., 2000; Gangopahyay et al., 2011), and protein kinase G (PKG) (Butt et al., 2000). Enzymatically, the phosphorylation of S1179 increases electron flow and calcium-calmodulin sensitivity (McCabe et al., 2000) which collectively increase NO synthesis at lower levels of intracellular calcium.

The phosphorylation of both S617 and S635 have also been shown to promote increased eNOS-derived NO release (Michell et al., 2002). The phosphorylaiton of S617 can be induced by PKA or Akt activity, and may serve to sensitize eNOS to calmodulin binding and modulate the phosphorylation of other eNOS sites (Michell et al., 2002; Bauer et al., 2003; Erwin et al., 2005). Mimicking the phosphorylation by mutating S617D only increases the Ca^2+^/CaM sensitivity without affecting overall enzyme activity (Michell et al., 2002). S635, in the FMN binding domain, is phosphorylated by PKA (Michell et al., 2002) and may represent a second stimulatory phosphorylation response (Boo et al., 2002). Mimicking phosphorylation with the S635D mutation results in enhanced eNOS overall activity as well as increased sensitivity to Ca^2+^/CaM. Both S617 and S635 are present on the same auto-inhibitory domain on eNOS. Deletion of this domain along with the other autoinhibitory domain containing S1179 results in an eNOS enzyme that is calcium-insensitive, which strongly support the ability of phosphorylation to modulate eNOS-calcium sensitivity (Church and Fulton, 2006).

The phosphorylation of T497 inhibits eNOS catalytic activity and is thought to interfere with the binding of calcium-activated calmodulin (Fleming et al., 2001). The phosphorylation of T497 is mediated by AMPK (Chen et al., 1999) and protein kinase C (PKC) (Chen et al., 1999; Fleming et al., 2001; Michell et al., 2001). Importantly, agonists such as bradykinin which increase NO release, simultaneously induce the dephosphorylation of T497 (Fleming et al., 2001; Harris et al., 2001), which enables calmodulin binding and eNOS activation. Dephosphorlyation of T497 is mediated by calcineurin and inhibited by cyclosporine A (Harris et al., 2001).

S116 was first identified as a phosphorylation site that can be induced by shear stress (Gallis et al., 1999). The impact of phosphorylation on S116 in the eNOS oxygenase domain remains controversial (Mount et al., 2007). S116 was previously suggested to be a negative regulatory site (Bauer et al., 2003). Evidence to support this derives from the ability of VEGF to induce the dephosphorylation of S116 and a phospho null mutation, S116A has increased activity (Kou et al., 2002). In contrast, mimicking the phosphorylation of eNOS by S116D mutation decreases basal NO release from endothelial cells and impairs endothelium-dependent relaxation in aortic rings (Li et al., 2007). The mechanism by which S116 phosphorylation impacts eNOS activity is not yet fully understood but may involve increased binding to the negative regulator, caveolin-1 [105]. Dephosphorylation is mediated by calcineurin which promotes increased activity via c-Src binding and phosphorylation of tyrosine 83 (Ruan et al., 2013). In contrast, shear stress (Gallis et al., 1999) and high density lipoprotein (HDL) (Drew et al., 2004) which increase eNOS activity have also been reported to increase S116 phosphorylation. Other studies have found no change in Ser116 phosphorylation with either shear stress or VEGF, which may reflect cell specific differences or greater methodological difficulty in detecting the phosphorylation of this site (Boo et al., 2002).

Y83 is a recently identified eNOS phosphorylation site (Fulton et al., 2005, 2008). Phosphorylation of this residue is mediated by Src kinase in response to different eNOS-activating agonists, which increases eNOS activity and NO production in both co-transfected COS-7 cells and in endothelial cells (Venema, 2002; Fulton et al., 2005). Phosphonull Y83F mutants of eNOS produce less NO and exhibit impaired endothelium-dependent relaxation when reconstituted in aorta from eNOS knockout mice. The tyrosine phosphorylation of eNOS has also been reported on Y659 by proline-rich tyrosine kinase 2 (PYK2). Phosphorylation of this site impairs eNOS activity (Loot et al., 2009).

### Protein-protein interaction

Calmodulin (CaM) was the first protein identified to directly bind and regulate the activity of eNOS. CaM binds to a cognate binding site on eNOS that lies between the oxygenase and reductase domains. Binding displaces an adjacent autoinhibitory loop and promotes NADPH-dependent electron flux to the heme moiety (Fulton et al., 2001). Electron transfer is impeded in the absence of bound calmodulin, thus eNOS catalytic activity is suppressed. eNOS activity is proportional to the level of intracellular calcium and the binding of calcium-activated calmodulin. The intracellular location of eNOS can influence its ability to respond to calcium, with eNOS at the PM being more responsive than intracellular sites particularly in unstimulated cells (Church and Fulton, 2006). This may be due to proximity to ion channels or transporters that are present in the PM. Prolonged cell stimulation and calmodulin binding may trigger the depalmitoylation of eNOS via Acyl-Protein Thioesterase-1 (APT-1) and membrane translocation (Michel et al., 1997; Yeh et al., 1999). While Ca^2+^/CaM is the primary means of activating eNOS in vitro, it is not the only game in town and this is revealed by changes in calcium-sensitivity and an ability to generate NO with resting levels of calcium. Many other factors can contribute to the Ca^2+^/CaM sensitivity of eNOS. For example, acylation, acetylation, phosphorylation, caveolin-1 and heat shock protein 90 (hsp90) binding can also influence the CaM-dependent activation of eNOS (McCabe et al., 2000; Fulton et al., 2001; Sessa, 2004).

Caveolin-1 (Cav-1) has been shown to directly bind to eNOS. The scaffolding domain of Cav-1 interacts with the caveolin binding motif on eNOS that is located between amino acids 350–358. The binding of Cav-1 inhibits eNOS activity and reduces NO production (Smart et al., 1999). Cav-1 binding is inhibited by calcium-mobilizing agonists and the elevation of intracellular calcium. Binding of calcium-activated calmodulin to eNOS displaces Cav-1 and facilitates eNOS activation (Govers et al., 2002; Fleming and Busse, 2003). Association of eNOS with Cav-1 can be decreased in a dose dependent manner by folic acid treatment (Taylor et al., 2013). Over-expression of Cav-1 in COS-7 cells suppresses NOS activity (Garcia-Cardena et al., 1997; Michel et al., 1997) and peptides encompassing the caveolin-1 scaffolding domain inhibit NO release from eNOS (Bucci et al., 2000). In Cav-1^−/−^ mice, both basal and stimulated eNOS activity and vasorelaxation are enhanced in blood vessels (Drab et al., 2001; Razani et al., 2001). In addition, peptides that displace Cav-1 from eNOS, enhance the synthesis of NO and promote vasodilation and further validate the inhibitory role of caveolin-1 in eNOS-dependent NO release (Bernatchez et al., 2011).

The hsp90s comprise a family of molecular chaperones responsible for the proper folding and maturation of client proteins (Pratt, 1997). As part of their chaperone activities, hsp90 regulates a variety of signal transduction pathways. Hsp90 has been shown to interact with eNOS under resting conditions and binding to eNOS is increased with numerous endothelial cell stimuli including VEGF, histamine, fluid shear stress, and estrogen, which promotes increased eNOS activity and NO release (Venema et al., 1996; Garcia-Cardena et al., 1998; Russell et al., 2000). There are multiple mechanisms by which hsp90 influences eNOS. Hsp90 binding to eNOS induces a conformational change in eNOS that promotes increased activity and increased enzymatic fidelity (Pritchard et al., 2001; Ou et al., 2003, 2004). Hsp90 binds to the oxygenase domain of eNOS between amino acid 310–323 (Xu et al., 2007) and it likely thus influences the binding/function of heme as has been shown for other heme containing proteins (Billecke et al., 2004; Ghosh and Stuehr, 2012). In addition, hsp90 can also function as a scaffold or platform for the recruitment and regulation of other regulatory proteins including kinases and phosphatases that can then secondarily influence eNOS function (Fulton et al., 2001). Geldanamycin (GA) can disrupt hsp90-eNOS binding and prevent Akt recruitment to eNOS to reduce eNOS activity (Roviezzo et al., 2007). Co-Immunopercipitaion revealed that interaction of hsp90 and eNOS was increased by folic acid (Taylor et al., 2013).

eNOS localization and activity can also be regulated by protein:protein interaction. Nitric oxide synthase interacting protein (NOSIP) and nitric oxide synthase traffick inducer (NOSTRIN) are both eNOS associated proteins. NOSIP and NOSTRIN, promote the translocation of eNOS from plasmalemmal caveolae to other intracellular compartments, such as Golgi. By promoting a reduced proportion of eNOS at the PM portion, NOSIP and NOSTRIN decrease eNOS activity and NO release (Dedio et al., 2001; Zimmermann et al., 2002).

Several transmembrane receptors and ion channels have also been shown to impact eNOS regulation. The bradykinin (BK) B2 receptor is a G-protein coupled receptor (GPCR) also functions as an allosteric regulator of eNOS activity. The binding of eNOS and the BK B2 receptor is dynamic and driven by Ca^2+^/CaM secondarily to cell stimulation with BK or other calcium mobilizing agonists and are reversed by blocking the elevation in intracellular calcium (Fulton et al., 2001). Using Coimmunoprecipitation experiments followed by mass spectrometry, the voltage dependent anion channel-porin was identified as a direct binding partner of eNOS and interaction with eNOS augmented activity probably through increased intracellular calcium (Sun and Liao, 2002).

Recently, another novel form of eNOS regulation was revealed, through phosphorylation mediated-protein association. The interaction of Pin1 prolyl isomerase with eNOS was observed only when S116 is phosphorylated. In both endothelial cells and blood vessels, inhibition of Pin1 increased NO release, and overexpression of Pin1 supressesed NO production, validating the functional significance of this interaction (Ruan et al., 2011).

eNOS function can also be indirectly regulated by many other proteins. APT-1 induces the depalmitoylation and translocation of eNOS (Yeh et al., 1999; Prabhakar et al., 2000). The cationic amino acid transporter-1 (CAT-1) is a major L-arginine transporter in ECs and has been shown to interact directly with eNOS and enhance its activity through a mechanism that paradoxically does not involve L-arginine transport (Li et al., 2005). In addition to CAT-1, the arginine recycling enzymes argininosuccinate lyase (ASL), and argininosuccinate synthase (ASS) have been shown to bind and regulate eNOS activity, but surprisingly this also does not require catalytic activity (Chen et al., 2013). The cell division cycle 37 (Cdc 37), a co-chaperone of hsp90 interacts directly with eNOS and inhibits its activity (Harris et al., 2006). The C-terminal hsp70-interacting protein (CHIP) associates with both hsp70 and 90, and negatively regulates eNOS trafficking into the Golgi complex (Jiang et al., 2003). Dynamin-2, a large GTPase has also been shown to bind to eNOS. Binding to eNOS can be increased by calcium ionophore and augments eNOS activity (Cao et al., 2001). Beta-actin, generally acknowledged for its house keeping functions, is associated with the eNOS oxygenase domain and that binding activates eNOS to increase NO production and decrease superoxide formation (Kondrikov et al., 2010). Another novel eNOS partner is the G-protein-coupled receptor kinase interactor-1 (GIT1) shown to bind eNOS in sinusoidal endothelial cells. Association of GIT1 with eNOS promoted Ser1179 phosphorylation, enzyme activation, and NO synthesis (Liu et al., 2012). In addition, eNOS has also been shown to interact with signaling molecules including Src kinase (Fulton et al., 2005), Akt-kinase (Michell et al., 1999), and sGC (Venema et al., 2003). Given the large array of eNOS interacting proteins an important unresolved question is how binding is coordinated and whether binding is direct or indirect. For example, hsp90 binds numerous co-chaperones and hundreds of client proteins and could readily mediate indirect associations of multiple proteins. Some binding partners are bound constitutively and other dynamic. The best described dynamic partner is calmodulin which may actually be bound constitutively and alternate between low and high affinity binding. How the other eNOS binding proteins are shuttled on and off is less well described.

### Substrate and cofactor availability

L-arginine is the substrate for eNOS and the catalytic activity also requires NADPH and the co-factor, tetrahydrobiopterin (BH4). Many studies have suggested that cellular deficiency of either L-arginine or BH4 can cause endothelial dysfunction by “uncoupling” eNOS. Uncoupled eNOS is a term used to describe a change in the ratio of NO to O^−^_2_ produced in favor of decreased NO and increased O^−^_2_. The consequences of this are both reduced synthesis and bioavailability of NO and increased levels of superoxide and peroxynitrite (Forstermann and Munzel, 2006). Depletion of the NOS substrate L-arginine has been proposed to occur via catabolism by arginase. Both arginase I and II in endothelial cells have also been proposed to inhibit eNOS activity via this mechanism (Wu and Morris, 1998; Zhang et al., 2001; Hallemeesch et al., 2002; Berkowitz et al., 2003). Increased Arginase II levels co-presents with endothelial dysfunction and has been observed with CVDs such as atherosclerosis (Ming et al., 2004) or hypertension (Zhang et al., 2004). Not surprisingly, the supplementation of L-arginine has been shown to have beneficial effects on eNOS activity (Elms et al., 2013) and in humans with pathophysiological conditions including hypercholesterolemia and hypertension. However, this remains a controversial approach as the levels of L-arginine, even in disease states, are much higher than required for eNOS synthesis (Drexler et al., 1991; Rossitch et al., 1991; Imaizumi et al., 1992) and more importantly long term supplementation of L-arginine may be detrimental (Chen et al., 2013).

Oxidation and depletion of BH4 levels promotes eNOS (Vasquez-Vivar et al., 1998) and endothelial dysfunction (Shinozaki et al., 1999; Hong et al., 2001). Supplementation of BH4 has been shown to improve endothelial-dependent vasodilation in animal models of diabetes (Pieper and Siebeneich, 1997) and insulin resistance (Shinozaki et al., 2000), as well as in patients with hypercholesterolemia (Stroes et al., 1997), diabetes mellitus (Pieper, 1997), essential hypertension (Higashi et al., 2002), and in chronic smokers (Heitzer et al., 2000). These findings suggest that limited synthesis or recycling of BH4 is an important rate-limiting step in the eNOS-dependent synthesis of NO in CVD states.

### S-nitrosylation

Not only is eNOS activity influenced by a wide range of protein regulators, but eNOS is itself post-translationally modified by S-nitrosylation in a product feedback relationship that constrains further NO synthesis. S-nitrosylation is a covalent modification of protein cysteine thiols by NO to yield an S-nitrosothiol (SNO) (Erwin et al., 2005; Lima et al., 2010). In endothelial cells, the source of NO for nitrosylation comes primarily from eNOS. The subcellular targeting of eNOS to the PM has been shown to be important for eNOS S-nitrosylation which is not surprising given the higher amount of NO produced at this location (Erwin et al., 2006). In quiescent or unstimulated endothelial cells, eNOS is predominantly S-nitrosylated on Cys-94 and Cys-99 (Dudzinski et al., 2006). Agonist stimulation promotes the rapid denitrosylation of eNOS and this occurs within a similar time frame to increased phosphorylation at Ser-1179 (Erwin et al., 2005). The nitrosylated cysteines are present within the zinc tetrathiolate cluster, a structure that is intimately connected with the eNOS dimer interface, but mutation of these sites does not impact dimer formation in intact cells (Erwin et al., 2005). Thus, the mechanism of exactly how nitrosylation represses the activity of eNOS remains poorly understood.

### Protein acetylation

The ability of aspirin and acetylating analogs to activate eNOS was due to direct acetylation of eNOS protein (Taubert et al., 2004). Deacetlyation of eNOS, acetylated at lysine 609 is mediated by histone deacetylase 3 (HDAC3), which decreases NO production by reduced calmodulin association (Jung et al., 2010). Sirtuin1 (SIRT1) also has been shown to regulate eNOS acetylation and inactivation of Sirt1 by oxidants can increase eNOS acetylation (Arunachalam et al., 2010; Donato et al., 2011). eNOS is also nitrated on multiple tyrosines (Zickus et al., 2008) and peroxynitrite inactivates eNOS by inducing uncoupling (Zou et al., 2002).

In summary, NO is a highly diffusible, gaseous signaling molecule that influences organ function by a number of different mechanisms. The synthesis of NO in the cardiovascular system is highly regulated through a complex array of transcriptional and post-translational modifications.

NO-dependent signaling becomes corrupted in CVDs states and occurs alongside progressive vascular dysfunction. The extensive knowledge on eNOS regulation, as detailed in this review, will enable a more insightful identification of variables that can be modified to restore NO balance in CVD states.

### Conflict of interest statement

The authors declare that the research was conducted in the absence of any commercial or financial relationships that could be construed as a potential conflict of interest.
